# Natural locomotion based on a reduced set of inertial sensors: Decoupling body and head directions indoors

**DOI:** 10.1371/journal.pone.0195191

**Published:** 2018-04-05

**Authors:** Ernesto de la Rubia, Antonio Diaz-Estrella, Arcadio Reyes-Lecuona, Alyson Langley, Michael Brown, Sarah Sharples

**Affiliations:** 1 Universidad de Málaga, Escuela Técnica Superior De Ingeniería de Telecomunicación, Departamento de Tecnología Electrónica, Campus de Teatinos, Málaga, Spain; 2 Human Factors Research Group, Faculty of Engineering, University of Nottingham, Nottingham, United Kingdom; University of Rome, ITALY

## Abstract

Inertial sensors offer the potential for integration into wireless virtual reality systems that allow the users to walk freely through virtual environments. However, owing to drift errors, inertial sensors cannot accurately estimate head and body orientations in the long run, and when walking indoors, this error cannot be corrected by magnetometers, due to the magnetic field distortion created by ferromagnetic materials present in buildings. This paper proposes a technique, called EHBD (Equalization of Head and Body Directions), to address this problem using two head- and shoulder-located magnetometers. Due to their proximity, their distortions are assumed to be similar and the magnetometer measurements are used to detect when the user is looking straight forward. Then, the system corrects the discrepancies between the estimated directions of the head and the shoulder, which are provided by gyroscopes and consequently are affected by drift errors. An experiment is conducted to evaluate the performance of this technique in two tasks (navigation and navigation plus exploration) and using two different locomotion techniques: (1) gaze-directed mode (GD) in which the walking direction is forced to be the same as the head direction, and (2) decoupled direction mode (DD) in which the walking direction can be different from the viewing direction. The obtained results show that both locomotion modes show similar matching of the target path during the navigation task, while DD’s path matches the target path more closely than GD in the navigation plus exploration task. These results validate the EHBD technique especially when allowing different walking and viewing directions in the navigation plus exploration tasks, as expected. While the proposed method does not reach the accuracy of optical tracking (ideal case), it is an acceptable and satisfactory solution for users and is much more compact, portable and economical.

## Introduction

In 1965, Ivan Sutherland presented the concept of The Ultimate Display [1] system that would control the existence of matter in a way that, for example, would allow us to take a seat in a virtual chair. This work has led to the consideration of the ideal virtual reality (VR) system. This is a fascinating concept with huge potential. An ideal VR system offers the possibility of experiencing any sensation, acquiring any knowledge, developing any skill and enjoying whatever we are able to imagine without limitations. Natural locomotion is a key to enabling advancement towards the realization of Ivan Sutherland’s vision. Natural locomotion attempts to approximate real-world walking and brings important benefits to virtual reality systems such as better proprioceptive cues, enhanced distance judgment and increased sense of presence [2]. For this reason, significant efforts and resources have been dedicated to developing locomotion interfaces during recent decades. Examples include flat treadmills [3–5]; spherical treadmills [6]; robotic tiles [7]; robotic actuators [8,9]; motorized skates [10]; and sliding disks [11–13]. These systems are able to let the user walk in a natural way while they are confined within a restricted space (walking in place systems). However, they all suffer from undesired effects such as unexpected inertial forces, disturbance when walking across non-planar surfaces or other issues derived from each particular approach. A different strategy to include natural locomotion in virtual reality systems allows the users to freely walk within large areas while tracking their movements (real walking systems). The main drawback of this approach is the need for a clear space that must be large enough to ensure that the users are not going to collide with any object. However, a small area can also be useful and sufficient for the development of a number of applications.

Another difficulty related to real walking systems is the need for high accuracy tracking capabilities over a wide area. Any tracking system should at least be able to track the position and the orientation of the head to ensure that the correct visual scene is displayed. In addition, a head mounted display (HMD) provides a virtual screen that surrounds the user and is convenient for the development of natural locomotion in virtual reality systems. The cable of the HMD is an issue that can be solved by carrying a laptop in a backpack or using a wireless video link that broadcasts images from the main rendering station to the HMD. It is important to highlight that in virtual reality experiences, there can be significant differences between the real and virtual movements of the user without these differences being noticeable to the user. The tolerable limits to this difference in terms of virtual/real travelled distance and turns have been determined in several studies [14–16]. For instance, it was determined [16] that the virtual distance travelled can be increased by up to 26% and reduced by up to 14% relative to the real distance without the user noticing this discrepancy. Aiming to relieve the imposed restrictions on the available space and the tracking system, Razzaque [17] proposed a technique, known as redirected walking, that exploits the mentioned discrepancies. According to his method, the position of the point of view in the virtual environment is manipulated so that the user, without realizing it, will tend to go to the centre of the tracking region.

However, since the final trajectory of the user cannot be predicted in all cases, it is necessary to provide a way to handle exceptional situations in which the users are about to collide with objects or are going to leave the tracked area. Reorienting the users when they reach the limits of the tracked area is one of these strategies [18,19]. Peck, Fuchs and Whitton [20] used distractor elements to minimize the impact in the virtual experience that lead to the reorienting of the user. Some authors propose metaphors that include the limits of the tracking area in the virtual environment, so that navigation can occur without space constraints [21,22]. Using overlapping virtual spaces that are not visible simultaneously is another strategy that helps to enhance virtual experiences when the tracked area is limited [23–25].

Many virtual reality systems that involve real-walking have been developed [26–30]. Most of the works cited previously employ optical trackers, and none of them rely on inertial sensors exclusively to track the movements of the user. Inertial sensors can provide many benefits to virtual reality, and several motion capture systems have been developed using these sensors [31–36]. Inertial sensors can be small, light, and inexpensive [37]; additionally, wireless models are available, which contributes to more comfortable and compact virtual reality systems. One major advantage is their ability to operate without a surrounding infrastructure that provides absolute references for taking measures [31]. This contributes to the reduction of the overall system cost. These sensors can be integrated with HMD and wearable infrastructure to create portable virtual reality systems that allow the users to freely walk and navigate through virtual environments. In addition, the user can also use their hands to interact with virtual objects or other users [38]. Although inertial sensors are usually employed to track orientation, they can also operate as position trackers [39]. However, position estimations are affected by drift errors and current estimations are based on previously estimated positions (this procedure is known as dead reckoning).

Bachmann et al. [40] developed a virtual reality system in which inertial sensors are placed on each shoe to track the position of the user. To update the orientation of the point of view, another sensor is attached to the head. Since this is a dead reckoning approach, the estimated position of the user is affected by drift errors. Nevertheless, this is not problematic because there is no need to establish a correspondence between the virtual space and physical space. However, the user could eventually collide with an object. To avoid this, Bachmann et al. [40] use ultrasonic trackers. Vincent [41] employed a similar system and included redirected walking to avoid collisions in a multiuser system. The accuracy of the estimated distance in inertial tracking systems in which the sensor is placed on the foot is approximately 1% of the travelled distance [42].

Most MIMUs (Magnetic and Inertial Measurements Units) include three axis accelerometers, three axis magnetometers and three axis gyroscopes. The magnetometers are employed to measure the angle around the vertical axis (yaw angle). However, the magnetic field of the earth is strongly distorted indoors by ferromagnetic materials present in the structure of the buildings. For this reason, magnetometers are usually not used indoors, and consequently, yaw angle estimations are based on gyroscopes, which are prone to drift errors. The use of gyroscopes is satisfactory for the performance of basic navigation tasks where a single MIMU is placed on the head and determines the forward direction (GD, gaze directed). However, this approach does not work for more complex tasks that require the decoupling of the direction of the head from the body’s direction (DD; Decoupled Direction) because both measures are independent and can lead to incoherent situations. This is because the yaw angle estimations of the body and head are affected by different drift errors. For example, after using the system for some time under the DD mode, the user could attempt to make a step forward and perceive an unexpected displacement to the right. This problem, that we will define as look forward error, has an enormous negative impact on the experience and could make the user feel very confused and disoriented.

This article proposes an original technique called EHBD (Equalization of Head and Body Directions) based exclusively on MIMUs that solves the above-mentioned problem by detecting when the direction of the head and the direction of the body match. It is based on the difference in the yaw angles that provides two magnetometers located at the head and the shoulder. Both sensors are close to each other and far from the ground. Consequently, both magnetometers are affected by the distortion of the magnetic field in a similar way.

The EHBD technique is applied in a wireless virtual reality system with natural locomotion based on 4 MIMUs that track both feet, the head and the shoulder. To evaluate the performance of EHBD, an experiment is carried out in which two navigation tasks are considered (navigation only and navigation plus exploration). The experiment compares the gaze-directed and decoupled direction modes. In the remainder of this paper, the natural locomotion virtual reality system we have developed together with the technique proposed for addressing the drift errors in yaw angle are described in the Materials and methods section, which also provides a detailed description of the conducted experiments. The experimental results are discussed in the Results and Discussion section, and finally, conclusions and future work are stated.

## Materials and methods

### EHBD technique

**Fig 1:**
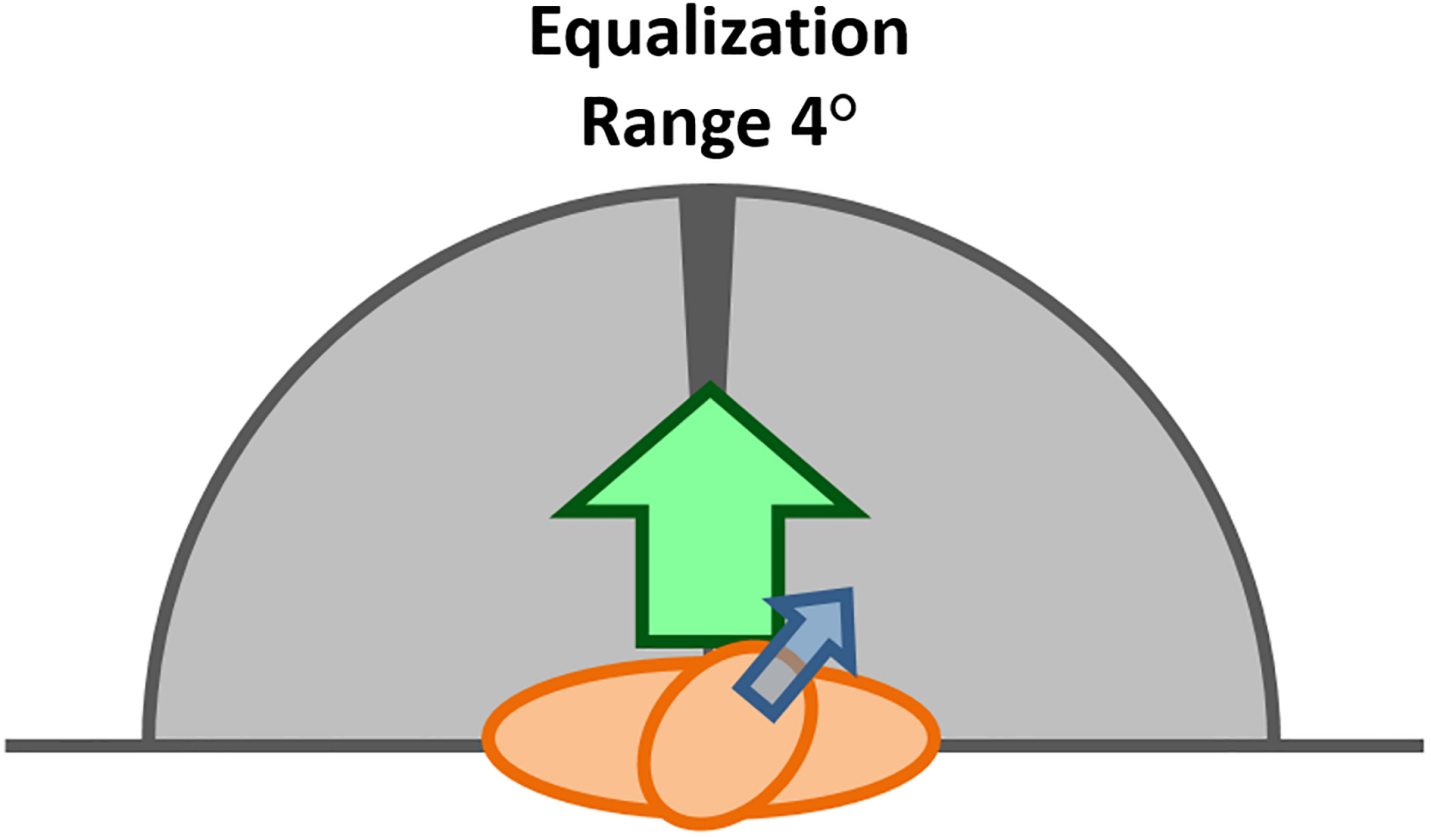
Body and head directions. Range of comparison of magnetic yaw angles to detect when the user is looking forward.

The EHBD (Equalization of head and body directions) technique corrects the look forward error described in the Introduction section. Although we do not use the magnetometers to estimate the absolute orientation of the sensors indoors, the EHBD technique uses them to detect when the user is facing forward. Specifically, the EHBD technique compares the magnetometer measurement results from the sensors on the head and the shoulder in each sample interval. When both magnetometers provide approximately the same magnetic yaw angle (Fig 1), the user is considered to look forward and the correction of the look forward error is then initiated. We refer to this correction process as equalization because it is a way to ensure that the body and head yaw angles of the gyroscopes are equal to each other. Since the user is more likely to notice any manipulation of the point of view rather than the changes in the forward direction, the equalization is carried out by modifying the forward direction only. For this, the inertial yaw angle of the body (provided by a gyroscope) is approximated progressively step by step to the value of the inertial yaw angle of the head at the beginning of the equalization.

**Fig 2:**
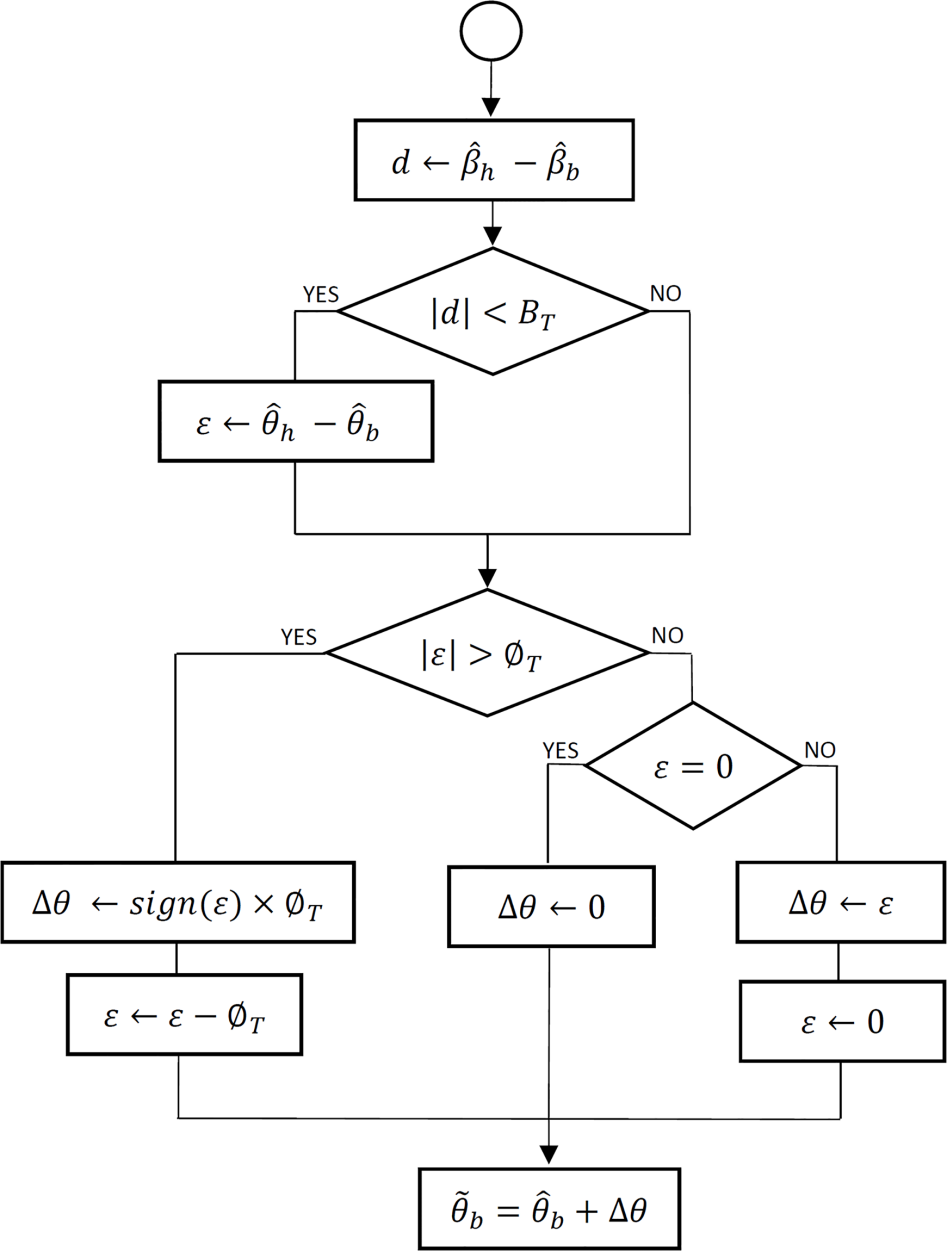
EHBD algorithm.

Fig 2 shows the EHBD algorithm in detail. For each sampling interval, we calculate the increase/decrease that must be applied in a progressive manner to the estimated inertial yaw angle of the body in order to eliminate the look forward error. First, the difference *d* between the magnetic head yaw angle, ${\hat{\beta }}_{h}$, and the magnetic shoulder yaw angle, ${\hat{\beta }}_{b}$, is calculated. If the absolute value of this difference is less than a certain threshold value *B*_*T*_, it is considered that a look forward state has occurred. In this case, the look forward error, defined as $\varepsilon ={\hat{\theta }}_{h}-{\hat{\theta }}_{b}$, where ${\hat{\theta }}_{h}$ and ${\hat{\theta }}_{b}$ are the estimated inertial yaw angles of the head and body, respectively, is calculated. Next, the increment/decrement value (step) Δθ is determined. If the absolute value of the look forward error ε is greater than a certain threshold value ∅_*T*_, the value *sign*(*ɛ*) × ∅_*T*_ is assigned to the step Δ*θ*, where *sign*(*x*) is the sign function of *x* (1, 0, -1 if *x* is greater than, equal to or less than 0, respectively) and the error value ε is updated by subtracting ∅_*T*_. Otherwise, when ε is not zero, the value of ε is assigned to step Δθ and then updated to 0. Finally, the corrected value of the estimated inertial yaw angle of body ${\tilde{\theta }}_{b}$ is set as ${\hat{\theta }}_{b}+\Delta \theta$.

According to this procedure, if the equalization takes place when the user is not walking, he or she will not notice any manipulation of the forward direction. On the other hand, if the equalization takes place when the user is walking, he or she may notice a slight change in the forward direction. However, according to our observations, these changes are typically unnoticeable. This is because equalization events occur frequently while the drift error in the inertial yaw angles increases very slowly. When the EHBD technique is applied, the drift error of the inertial yaw angles persists but does not cause any problem for the real-walking system because this drift error affects the estimations of inertial yaw angles for the head and the body in the same way.

A simulation (S1 File) has been carried out using a basic calibration error model [43] for a gyroscope where the estimated angular velocity $\hat{w}[k]$ of the sensor at time kΔt is defined as:

$$\hat{w}\left[k\right]=\;a+bw\left[k\right]$$ (1)

where *w*[*k*] is the true value and *a* and *b* are the offset and scaling errors, respectively. The estimated angle $\hat{\theta }\;[k]$ is obtained by integrating the estimated angular velocity $\hat{w}[k]$:

$$\hat{\theta }\;\left[k\right]=\;\hat{w}\left[k\right]\Delta t+\;\hat{\theta }\;\left[k-1\right];\;\hat{\theta }\;\left[0\right]=\;0$$ (2)

where Δt is the sampling interval. The true angular velocity *w*[*k*] can be modelled as a half-cycle of a sinusoidal signal with amplitude $\emptyset \frac{\pi \Delta t}{T}$, where *T* is the period of the signal and ∅ is the angle reached in a half cycle.

**Fig 3:**
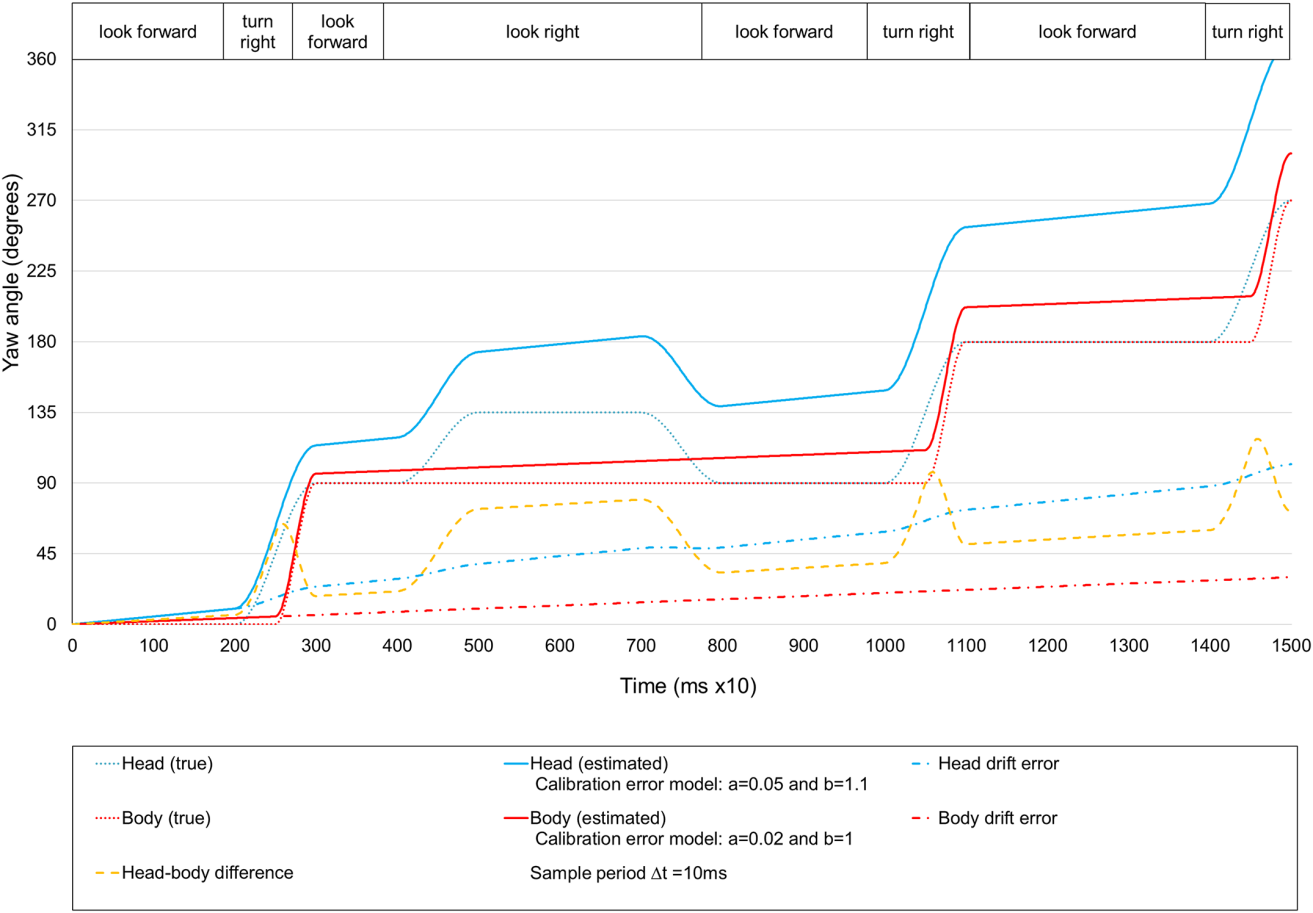
Simulation of a virtual walk. Evolution of head and body yaw angles and the generated drift errors.

The simulated trajectory is as follows (Fig 3): the user starts to walk straight looking to the front (look forward state) and a few seconds later turns 90 degrees to the right (turn right state). Then, the user continues to walk straight but for a few seconds turns his or her head about 45 degrees to the right (look right state). Then, he or she goes back to the look forward state. Later, he or she turns again by 90 degrees, goes straight and turns right again.

Fig 3 shows the estimated (solid lines) and true values (dashed lines) of the yaw angles for the head and body sensors. The drift errors of each sensor (dashed lines) and the difference between the estimated yaw angles of the head and body (dotted line) are also shown. Note that the head and body sensors have different displacement error values (*a*) and scale (*b*) and that they have been set to very large values to be able to observe the drift error well. The actual values are usually an order of magnitude smaller [44]. If the yaw values are true (ideal case), there are no drift errors and the head and body yaw angles differ only in two cases: in the turn right state because the head begins to rotate before the body, and in the look right state because the user turns the head but not the body. However, if the values are estimated (real case), drift errors are also different for each sensor. Therefore, in the look forward state, there is an undesired difference between the yaw angles of the head and body that disorients the user and that should be corrected. We refer to this undesirable difference as the look forward error.

**Fig 4:**
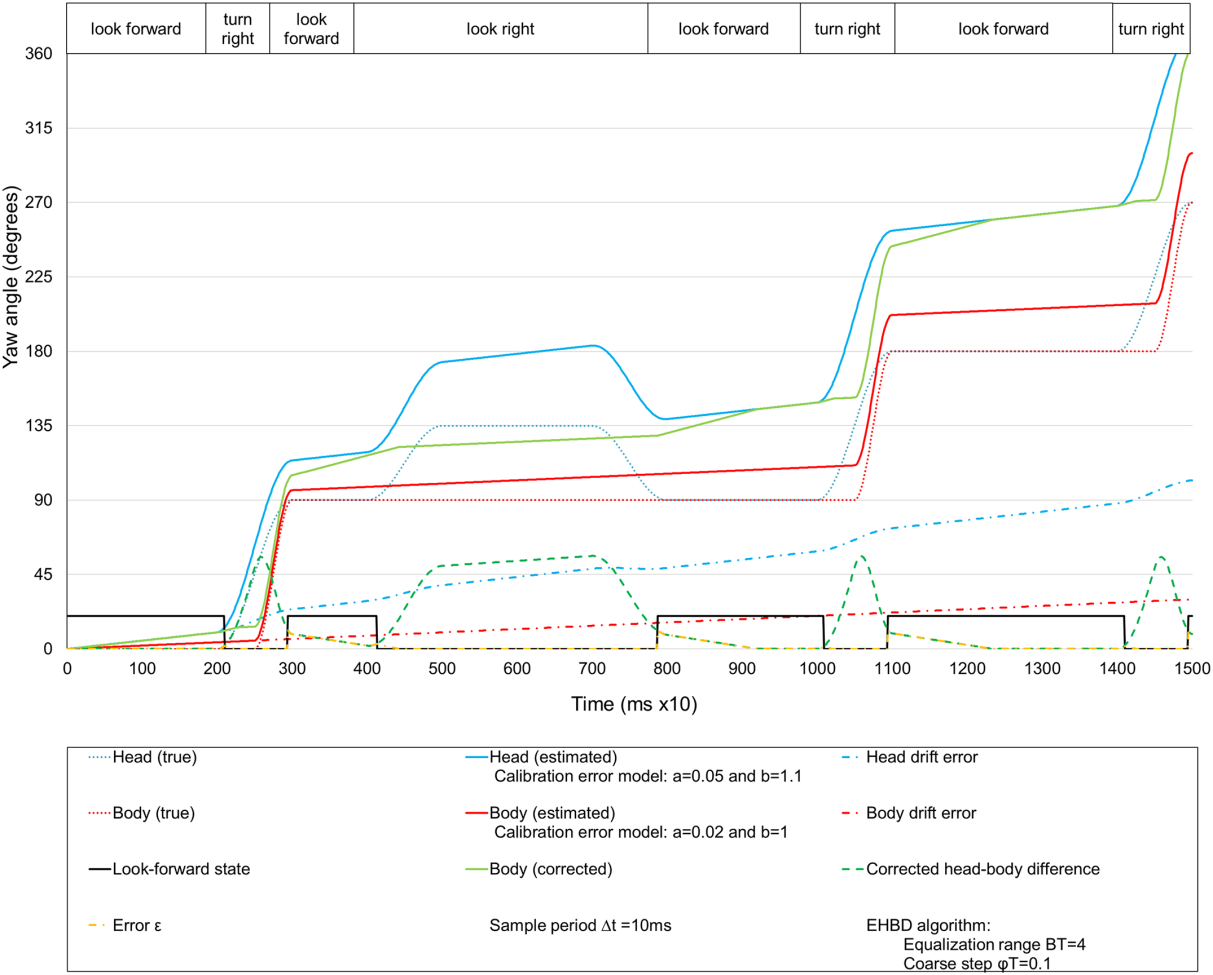
Simulation of a virtual walk with EHBD. Evolution of head and body yaw angles and drift errors when the EHBD technique has been applied.

The simulation shown in Fig 3 is repeated but with the EHBD algorithm activated, as shown in Fig 4. Note that when the look forward state is detected, the estimated yaw angle of the body is corrected progressively (step by step) until it reaches the value of the estimated yaw angle of the head at the beginning of the equalization. We note that this procedure does not eliminate the drift error but rather only the difference between the two inertial estimated angles when the user looks ahead.

The EHBD technique requires that the magnetic field gradient of the workspace be low so that the difference in the measurements of the magnetometers on the head and the shoulder is kept within a certain margin in order to correctly determine the look forward state. To achieve this, the workspace must be free of obstacles and the magnetometers must be at least 1.5 m [45] away from the elements (walls, floors and ceilings and any object or equipment) that can introduce hard and soft iron distortions, which increase the gradient of the magnetic field. In the tests performed in our laboratory, these conditions are fulfilled with the magnetic field differences between both magnetometers not exceeding 2 degrees. Before using the system, a calibration process must be performed in order to avoid the effect of misalignments of the magnetometers and possible distortions of the magnetic field caused by the equipment (HMD and back pack) that the user is wearing. For this purpose, the user must turn by 45° up to eight times to complete a lap. After each turn, the user must look forward and two samples are taken from the magnetic yaw angles: one for the head and another one for the shoulder. As a result of this procedure, a calibration table is obtained. Given the magnetic yaw angle of the body, this table provides the magnetic yaw angle of the head at which the user would be facing forward. The system applies this table continuously to determine when the user is facing forward, and the equalization of head and body directions is then applied.

### Experimental design

We have conducted an experiment to evaluate the EHBD technique that equalizes the directions of the head and the body. For this purpose, the DD mode in which this technique is applied is compared to the GD mode in this study. When GD is active, the walking and head directions are the same. Consequently, when the user walks, he advances in the direction of the head. On the other hand, the DD mode considers different directions for the body and the head. Thus, users can turn their head while walking without affecting the trajectory. GD is suitable for simple navigation tasks but not for complex tasks such as simultaneous navigation and exploration tasks, or curved trajectories (for example, entering through a doorway). In this case, the DD mode is more appropriate. Our hypothesis leads us to expect that users will match the target trajectory during the navigation task (NAV) under DD and GD conditions in a similar way. Furthermore, we expect that the DD configuration will outperform GD when exploration is taken into account in the navigation plus exploration task (NEX). Prior to the experiment, the ethics committee of Nottingham University’s Engineering Faculty reviewed and approved the experiment.

#### Participants

Twenty-nine participants participated in the experiment. Five (3 males, 2 females) decided to leave after a few minutes because they started to experience VR-induced sickness symptoms. Twenty-four participants completed the experiment (18 males, 6 females) matching the minimum number of subjects recommended by the power analysis (a large effect size was assumed, 0.4). Their ages range from 20 to 53 (mean (M) = 30, standard deviation (SD) = 9.1). All participants volunteered without receiving any form of compensation for participating in the experiment.

#### Apparatus

**Fig 5:**
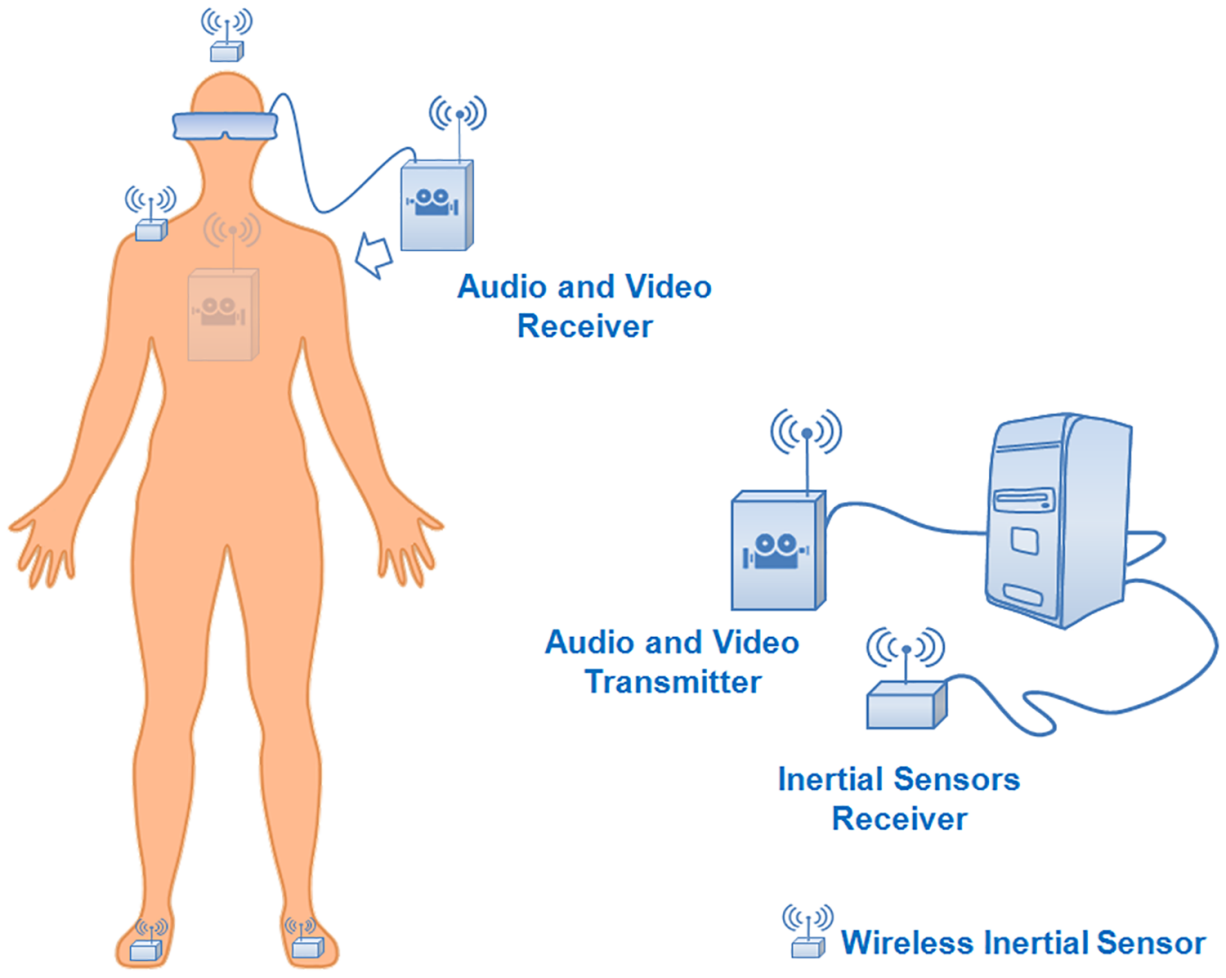
Platform. Main components of the experimental platform.

Fig 5 shows the main components of the experimental platform. The user carries an HMD and backpack that contains the video receiver together with its battery and wears wireless headphones and four InertiaCube 3 sensors from Intersense located on the feet, head and shoulder. The last two sensors are used to equalize the estimations of the yaw angles of the head and the body. The rendering station in combination with the receiver of the inertial sensors and the video link transmitter complete the system.

Regarding the technical specifications of the system, the wireless video link manufactured by Sensics has more than one hour of autonomy. Its operating range is 100 m with an end-to-end latency of 30 ms. It supports full HD video streams (1920x1080 pixels) at 60 Hz. The backpack is rather light. Its weight is around 1 kg, and it includes the video link receiver and its battery. The employed HMD is Oculus Rift (development kit 1) from Oculus VR. This HMD supports a resolution of 1280x800 pixels at 60 Hz. Its horizontal field of view is 90 degrees, and its latency is above 30 ms for a system running at 60 frames per second. This latency includes all the stages in the process chain including sensing, sending data, sensor fusion, virtual world simulation, rendering and video output. The HMD is complemented by wireless headphones to provide audio-visual feedback to the user. The rendering station generates stereoscopic images at 60 frames per second, which is the maximum frame rate that the HMD supports.

The four InertiaCube 3 sensors are used to track the movements of the user and operate at 110 Hz with a latency of 6 ms corresponding to the time interval between the time at which the measurement is performed by the sensor and the arrival of the data to the remote computer. Their maximum range is 30 m. These sensors include three axis accelerometers, three axis magnetometers and three axis gyroscopes. We did not use the raw output of the magnetometers. Instead, we use the estimation of the magnetic yaw angle provided by the InertiaCube 3 sensors obtained from the three-axis magnetometer embedded in each sensor. This magnetic yaw angle is provided by the software development kit of Intersense and is denoted as CompassYaw.

To track the movements of the feet, an inertial sensor is attached to each shoe. For this purpose, we developed a technique that improves the previous results in terms of accuracy and smoothness of the trajectory [46]. To estimate the position of the user, we consider relative increments of the position and orientation from each foot. The inertial sensor placed on the head provides its orientation. By combining these data, we can replicate the movement of the point of view in the virtual environment. We also add oscillating movements to enhance the feeling of walking. More details about the system can be found in de la Rubia and Diaz-Estrella [47]. We do not use the sensor embedded in the HMD. Rather, we obtain the body and head orientations from the same type of sensor in order to make them comparable. The EHBD algorithm uses the following values for its parameters: *B*_*T*_ = 2 degrees and ∅_*T*_ = 0.5 degrees.

**Fig 6:**
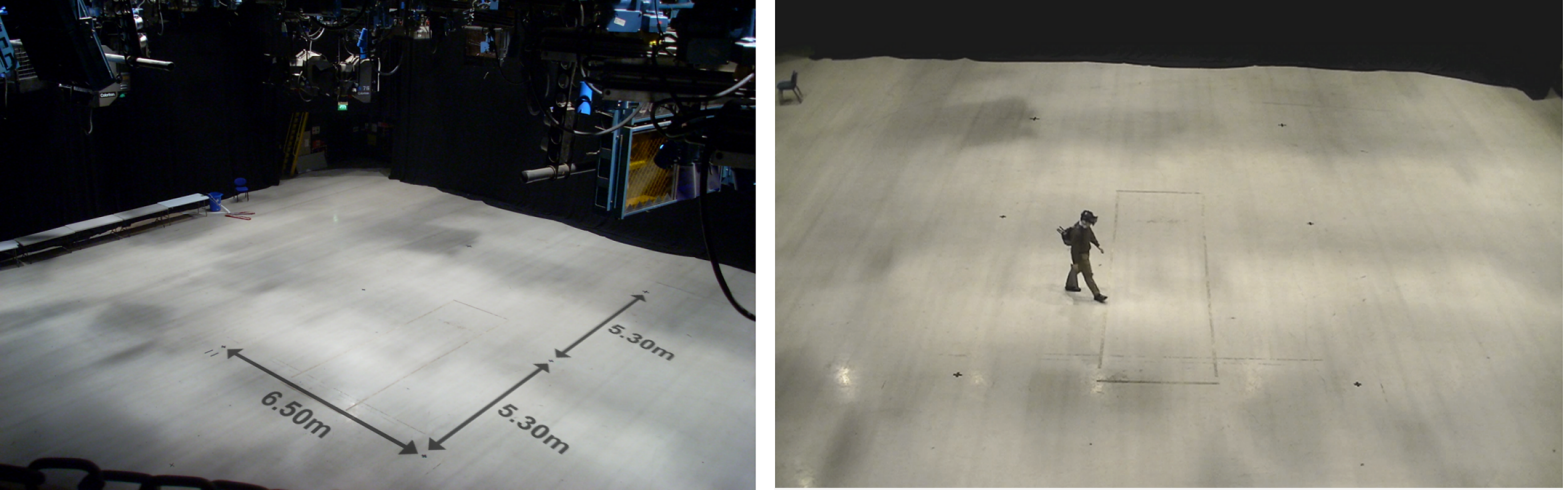
Experiment venue. Perspective view from the television studio where the experiment was performed (left). Subject using the system (right).

**Fig 7:**
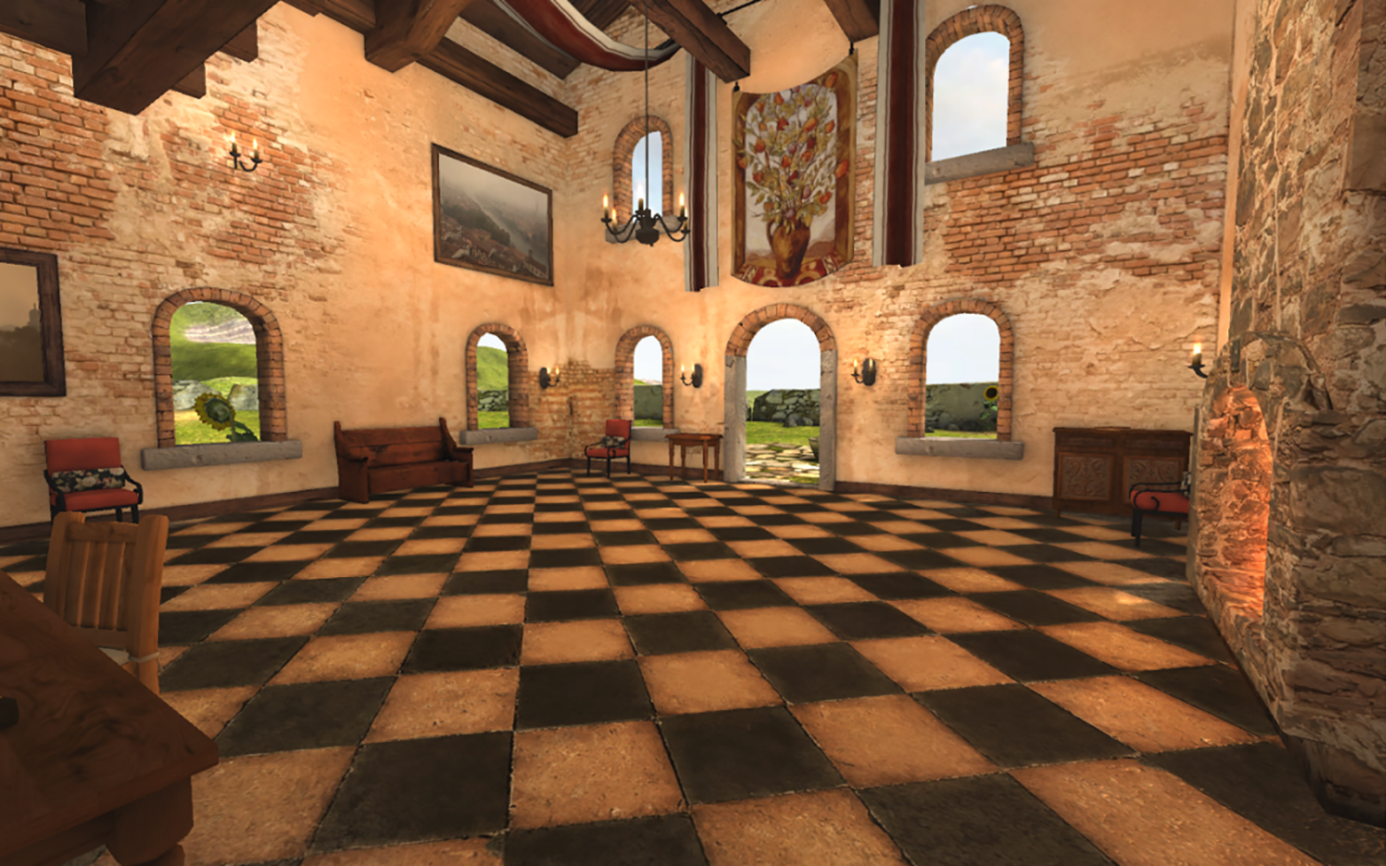
Employed virtual environment. Tuscany virtual cottage from Oculus VR after modification to perform the experiment.

**Fig 8:**
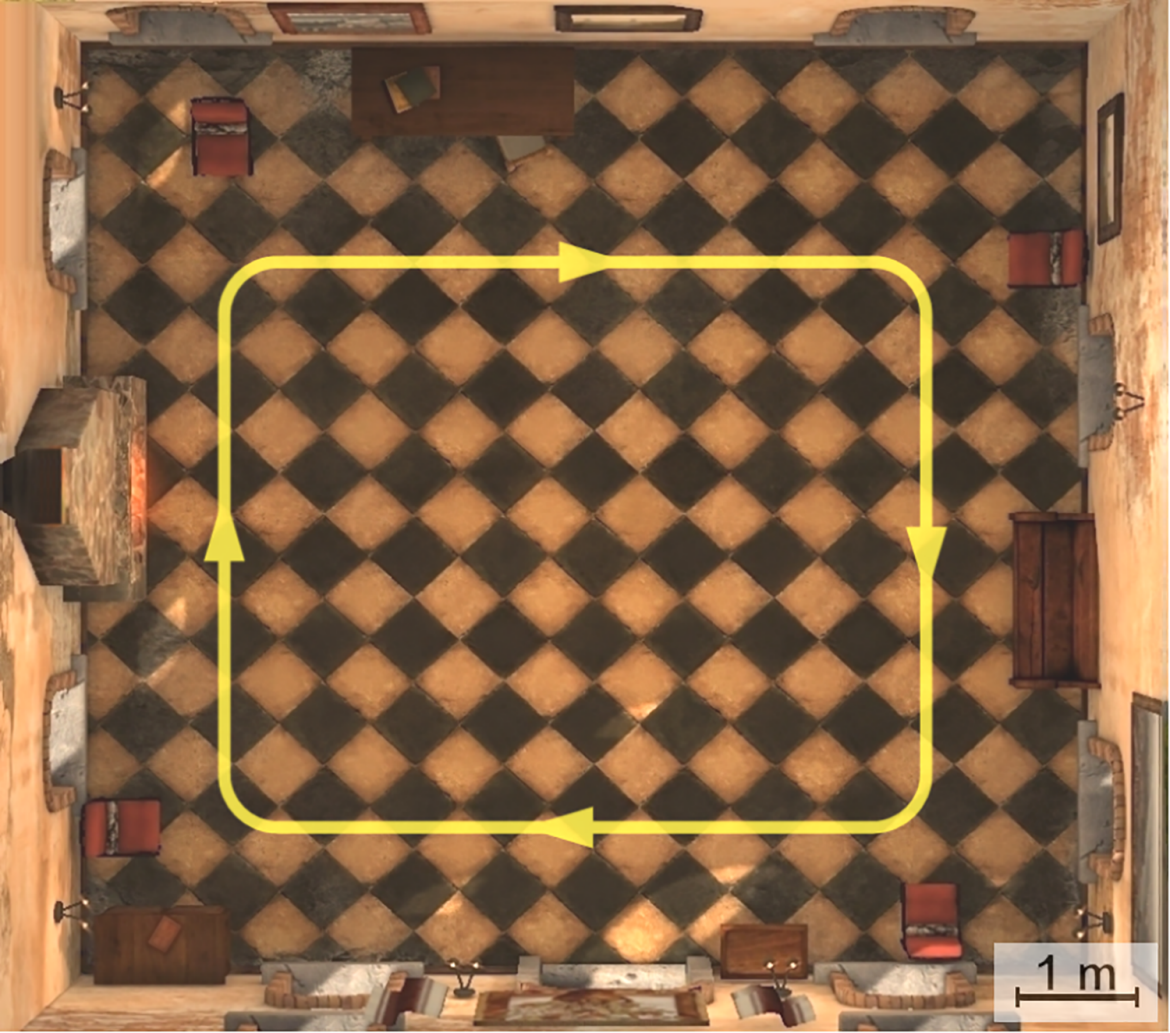
Target path. Target path that participants followed during the NAV and NEX tasks (lines and arrows are not displayed in the virtual environment).

The experiment was conducted in a television studio at the University of Nottingham. The studio is acoustically isolated, and its dimensions are 26 × 26 m (Fig 6). To perform the experiment, we modified the Tuscany virtual environment created by Oculus VR by removing the stairs and some furniture in order to let the participants walk across the entire room (Fig 7). To assist the participants during the navigation in both tasks (NAV and NEX), four chairs were placed close to the corners of the room. The target path that the subject followed was defined by these chairs (Fig 8) and was a rectangle with dimensions of 5.7 × 4.6 m. The travelled distance per lap is 20.6 m.

**Fig 9:**
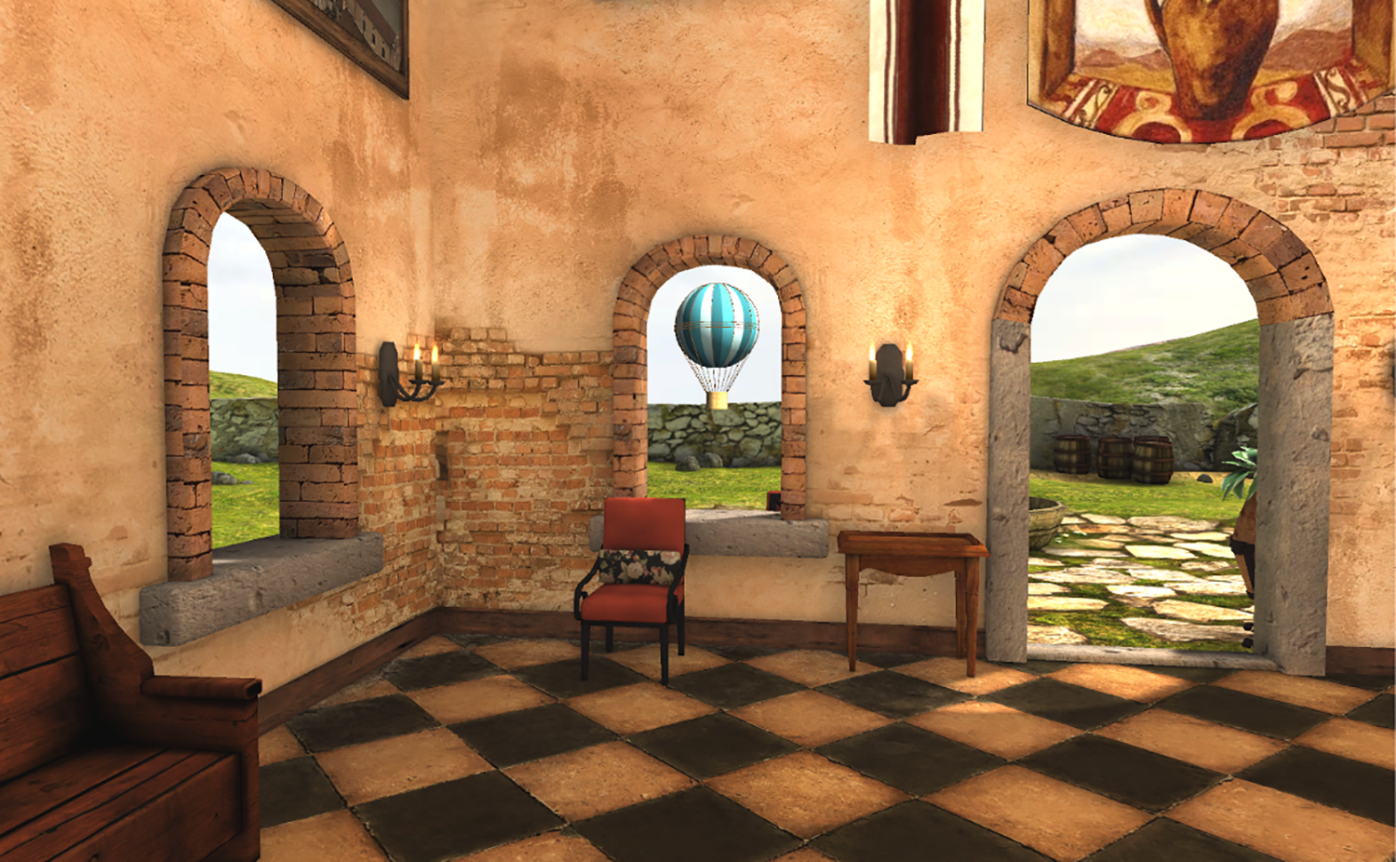
Animated balloons for exploration. Animated balloons that appear through the windows to include exploration in the experiment.

To include visual exploration within the virtual experience, we animated two balloons that appeared and disappeared through the windows of the cottage (Fig 9). Participants looked for the balloons during the NEX task. The windows at which the balloons appear follow a random sequence. The same sequence is applied to every participant so they all perform the experiment under the same conditions.

#### Procedure

We consider two tasks. The first one involves navigation only (NAV), and the second one adds exploration (NEX). Participants were randomly distributed into two groups, with either the GD or DD conditions. Each person performed both tasks, NAV and NEX (2 × 2 mixed-subjects design). For counterbalancing the results, half of the participants in each group performed the tasks in one order and the other half in the opposite order.

Prior to starting the experiment, the participants completed several forms and questionnaires: consent, demographic information, the 29-item version of the simulator sickness questionnaire (SSQ) proposed by Kennedy, Lane, Berbaum and Lilienthal [48] and a questionnaire to collect problems or particularities of the subject related to the sense of sight (SQ).

The sensors were attached to the shoes using Velcro straps before the remaining elements of the system were secured to the participants. Then, misalignment of the sensors on the head and the shoulder was calibrated by applying the procedure mentioned above in the EHBD technique section. The sensors on the feet also needed to be calibrated according to the procedure described in de la Rubia and Diaz-Estrella [47].

The participants then began the adaptation phase in which they practiced using the system by walking inside the cottage. This phase lasted as long as the participants required. Once they felt ready to start the experiment, the participants watched a video of a researcher performing the task with the video showing two views from the virtual and real environment simultaneously.

The participants then performed the task and were asked to go from one chair to the next without stopping. They were informed that the aim of the NAV task was to “complete as many laps as possible” and that the aim of the NEX task was “to complete as many laps as possible and to count aloud as many balloons as possible”. Participants were also told that both goals in the NEX task were equally important. The order in which each participant performed the tasks depended on the counterbalance subgroup to which they were allocated.

Each task was composed of three trials lasting 90 seconds each. Between two consecutive trials, the participants removed the HMD and rested for 2 minutes. Once the participant finished the third trial of task one, the cybersickness questionnaire was completed. Then, the second task was explained, performed and followed by the final completion of the sickness, usability, presence [49] and general aspects questionnaires (preference, positive and negative aspects of the system). The usability questionnaire is an adaptation from that employed in the European Project SATIN (Sound And Tangible Interfaces for Novel product design Project) and was specifically designed for the evaluation of virtual reality systems [50].

#### Statistical analysis

Our quality metric is the deviation of the ideal path. The rationale for this is as follows: if the EHBD technique were not applied, the drift error between the relative yaw angles estimated for the head and the body will grow. Values of around 45 degrees will make even walking a few steps difficult. This would be quite disturbing for the users because rather than advancing straight forward as they expect, they will follow a diagonal direction (45 degrees to the left or to the right). This would typically occur before reaching the 90 seconds that each session lasts. Therefore, a higher extent of control over the desired direction during locomotion as well as higher effectiveness of the proposed EHBD technique will be obtained for lower deviation from the ideal path. To obtain the deviation from the ideal path, we have calculated the root mean square distance from the estimated position to the closest point of the ideal path.

Independent-samples t-tests were employed to analyse the data collected in the experiment (deviation from the ideal path, completed laps and counted balloons) and the questionnaires (demographic information, SSQ, SQ, presence, usability and general aspects). All data and details of statistical analysis can be found in the S2 File.

## Results and discussion

**Fig 10:**
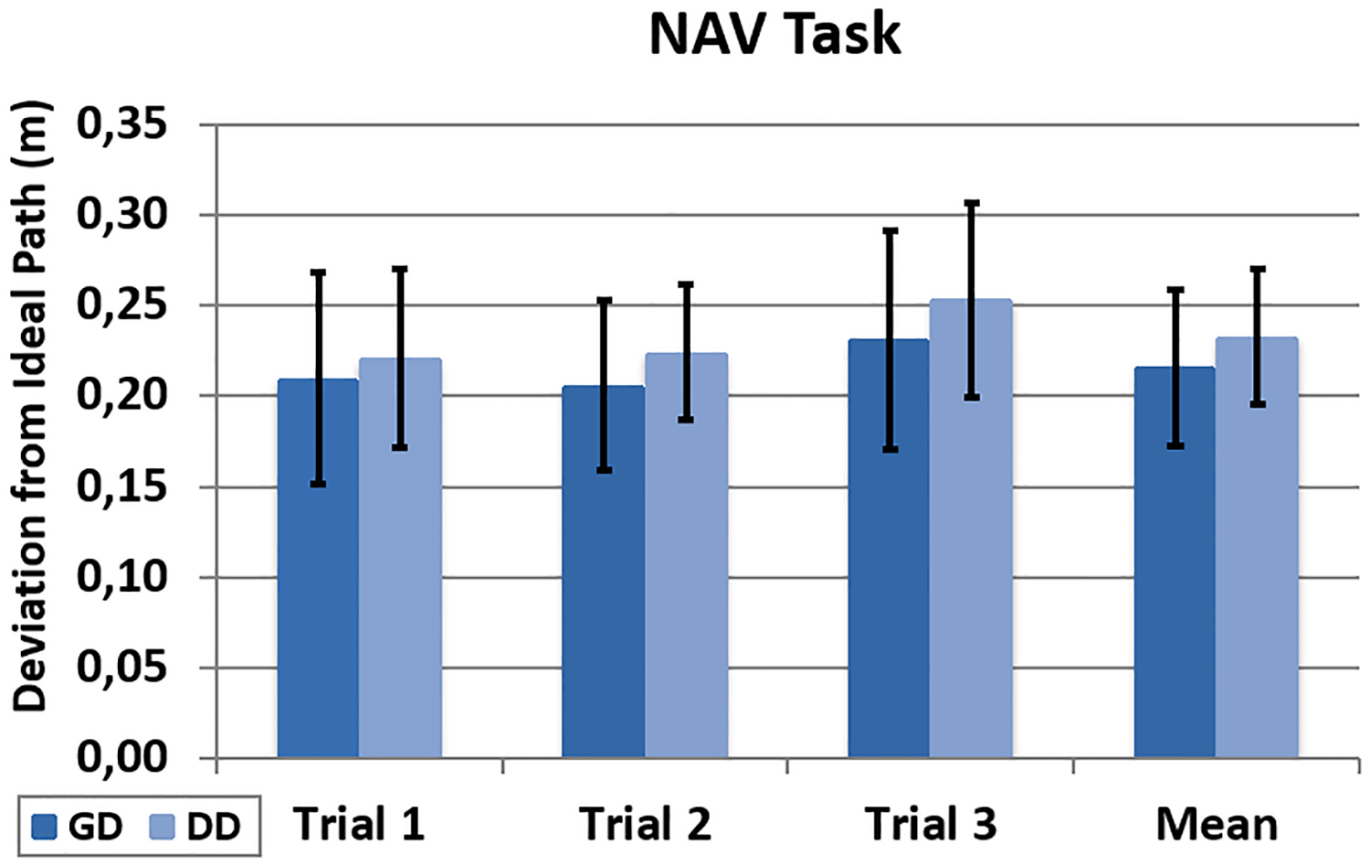
Deviation during the NAV task. Registered deviation from the ideal path during the NAV task across trials and the global mean.

The analysis showed that the deviation from the ideal path during the NAV task does not differ significantly between the DD and GD groups (Fig 10). The mean deviation for the three trials is 22 cm under the GD condition. This is 4% less than the mean of 23 cm measured for the DD group (t (22) = 1.07, p = 0.297).

**Fig 11:**
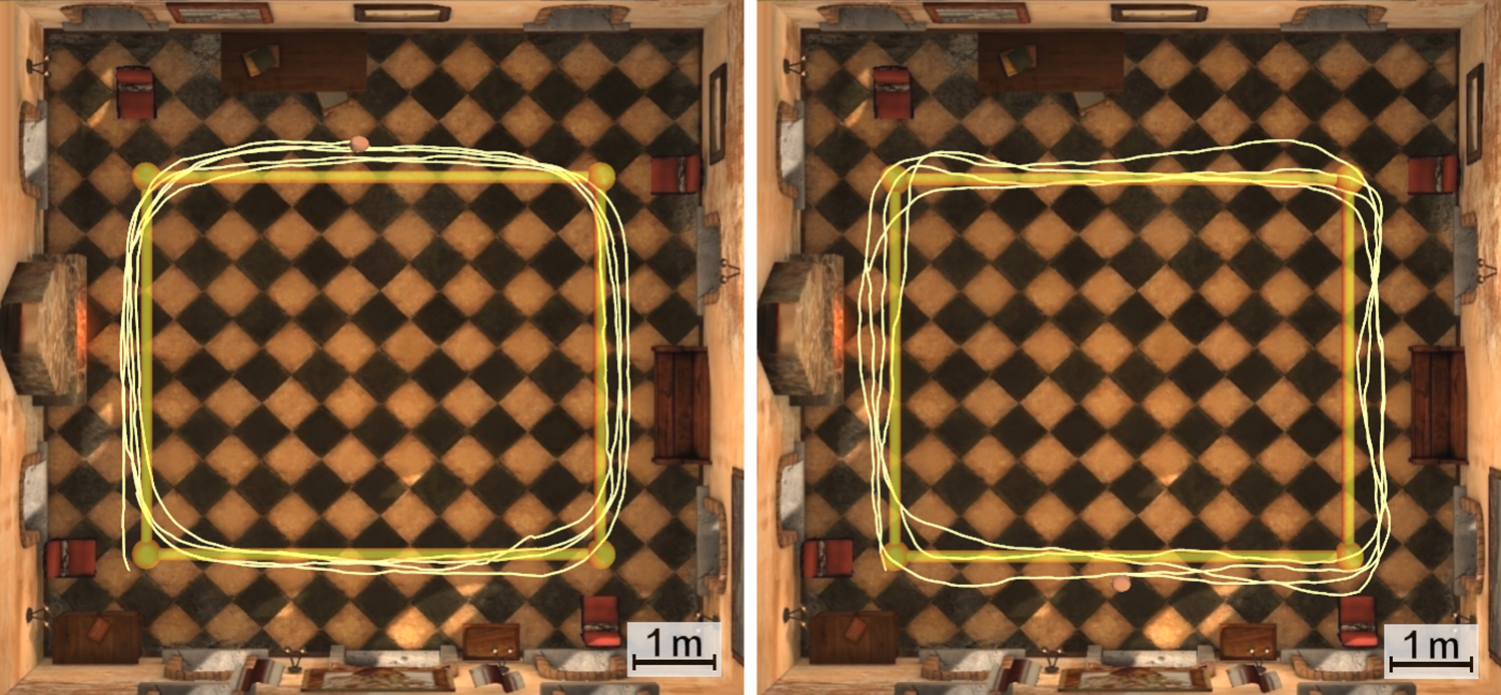
Sample trajectories (NAV). Sample trajectories of a trial under GD (left) and DD (right) conditions during the NAV task.

This result was expected as the GD mode provides a high degree of control of the trajectory when the users walk while looking forward. This is what typically occurs during the NAV task because the participants focus on the chair that is in front of them. The accuracy of the system during the NAV task is illustrated in Fig 11.

Slightly higher deviations for the DD condition were expected and can be observed in the NAV task. We consider this to be due to small errors in the estimated forward directions that arise during the equalization of the head and body directions. These errors are related to the threshold *B*_*T*_ (trade-off value), small deviations during the calibration process and the swing movement of the body in the yaw angle that occurs during walking. However, in spite of these errors, the deviation under the DD condition in the NAV task is small and the behaviour of the system is satisfactory in the GD and DD groups.

**Fig 12:**
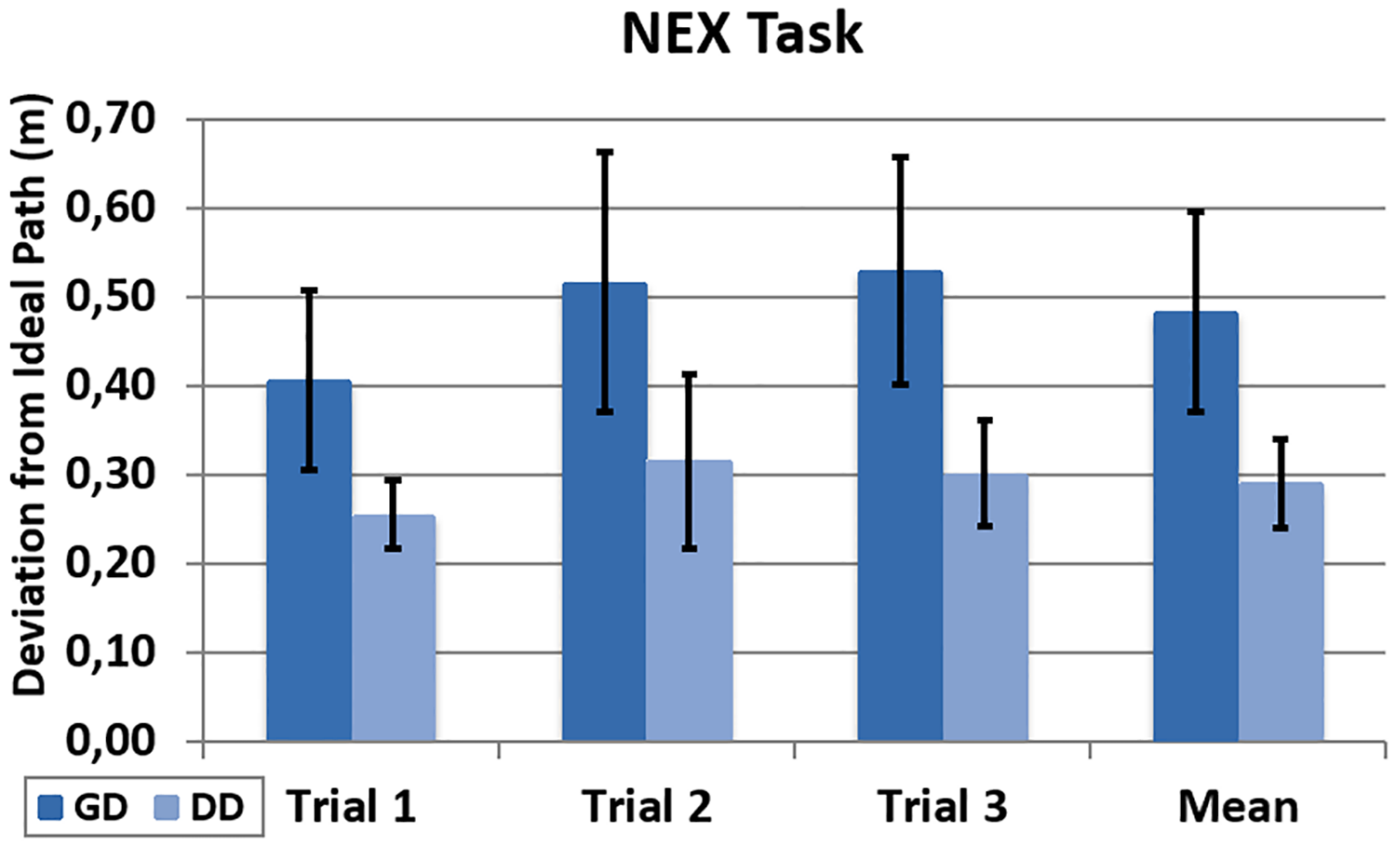
Deviation during the NEX task. Registered deviation from the ideal path during the NEX task across trials and the global mean.

When exploration comes into play in the NEX task, the DD mode outperforms GD as expected [51]. The mean deviation of the three trials increases up to 48 cm under the GD condition. This is 65% higher than the mean of 29 cm obtained in the DD group (Fig 12). This difference is statistically significant (t(15) = 5.48, p<0.001).

**Fig 13:**
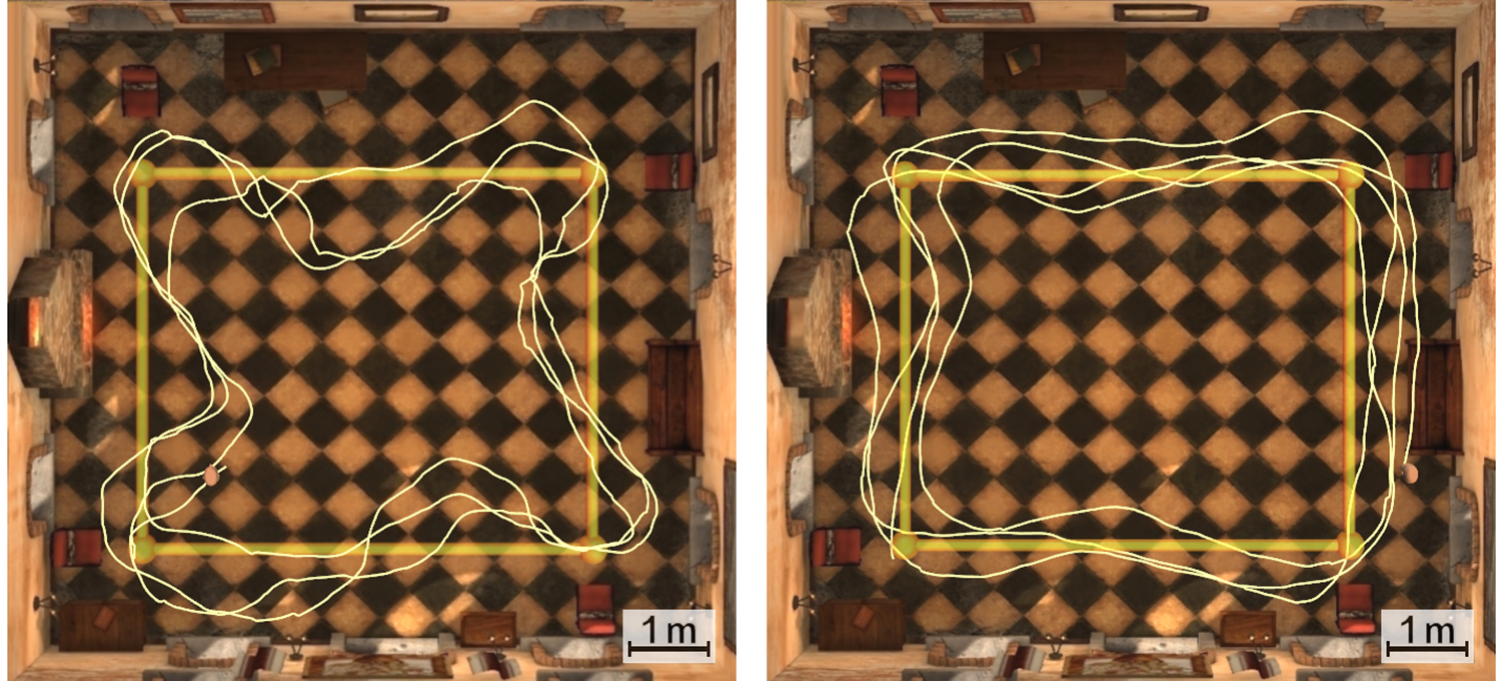
Sample trajectories (NEX). Sample trajectories of a trial under GD (left) and DD (right) conditions during the NEX task.

As the participants complete the laps while looking for balloons in the NEX task, the trajectory moves from the ideal path under the GD condition. On the other hand, participants in the DD group are able to more precisely control the trajectory of the point of view (Fig 13). This shows that the EHBD technique is working properly, confirming its validity to a certain extent.

**Fig 14:**
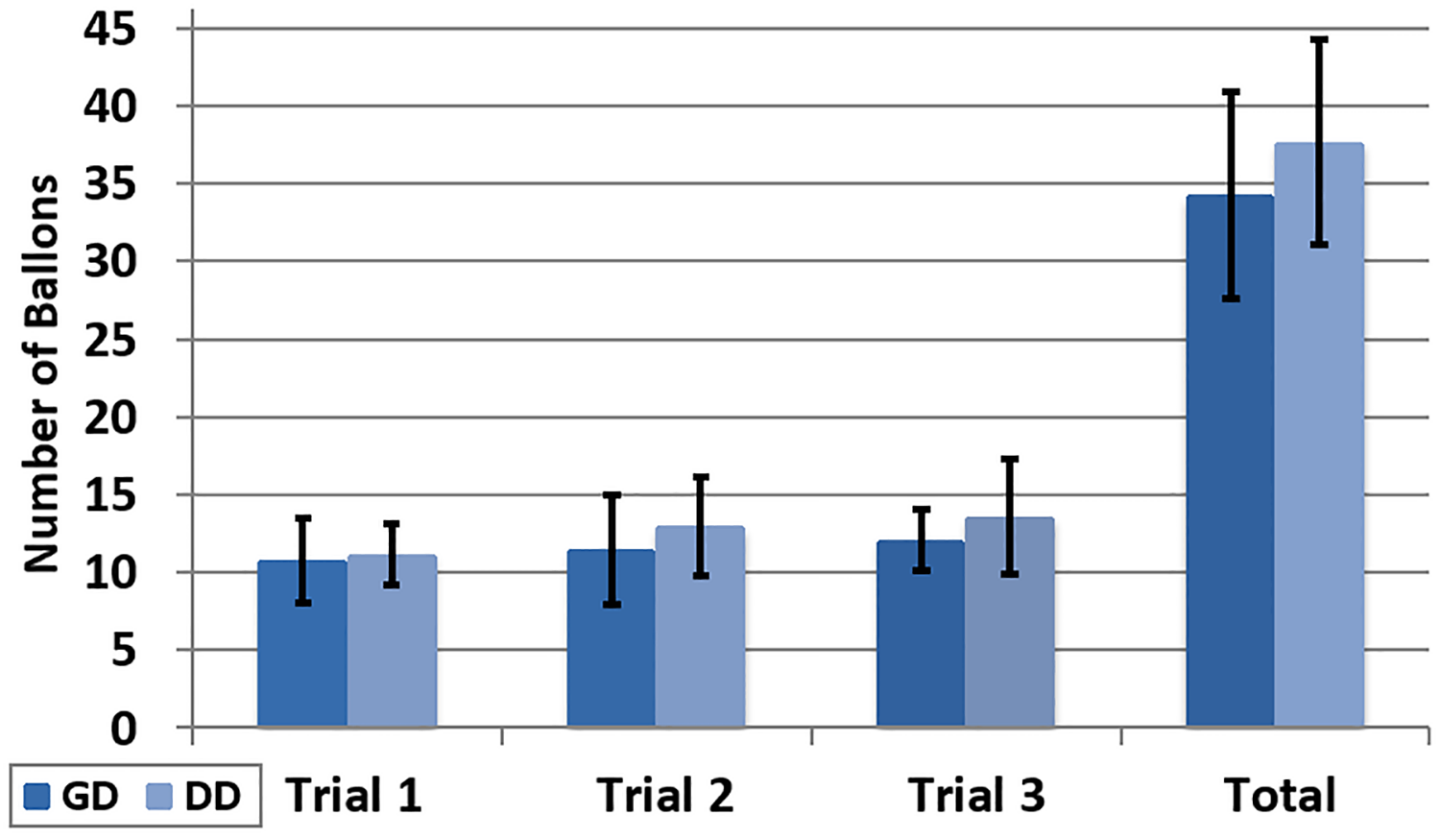
Counted balloons in the NEX. Number of balloons counted by participants during the NEX task across trials and the total value.

The difference in the number of balloons counted by the participants is not statistically significant (Fig 14). The mean value of the total number of balloons reported per participant under the DD conditions is 37.7. This is 10% higher than the 34.2 for the GD condition (t(22) = 1.26, p = 0.218).

Although statistical significance is not achieved, a positive and consistent relation across all trials can be observed in favour of the DD condition.

Higher levels of environmental validity in a navigation interface are likely due to the use of fewer cognitive resources, and hence, an increase in performance of the secondary task can be expected. The results suggest that participants felt more comfortable using the DD mode, which may cause a slight improvement in the performance while counting balloons.

**Fig 15:**
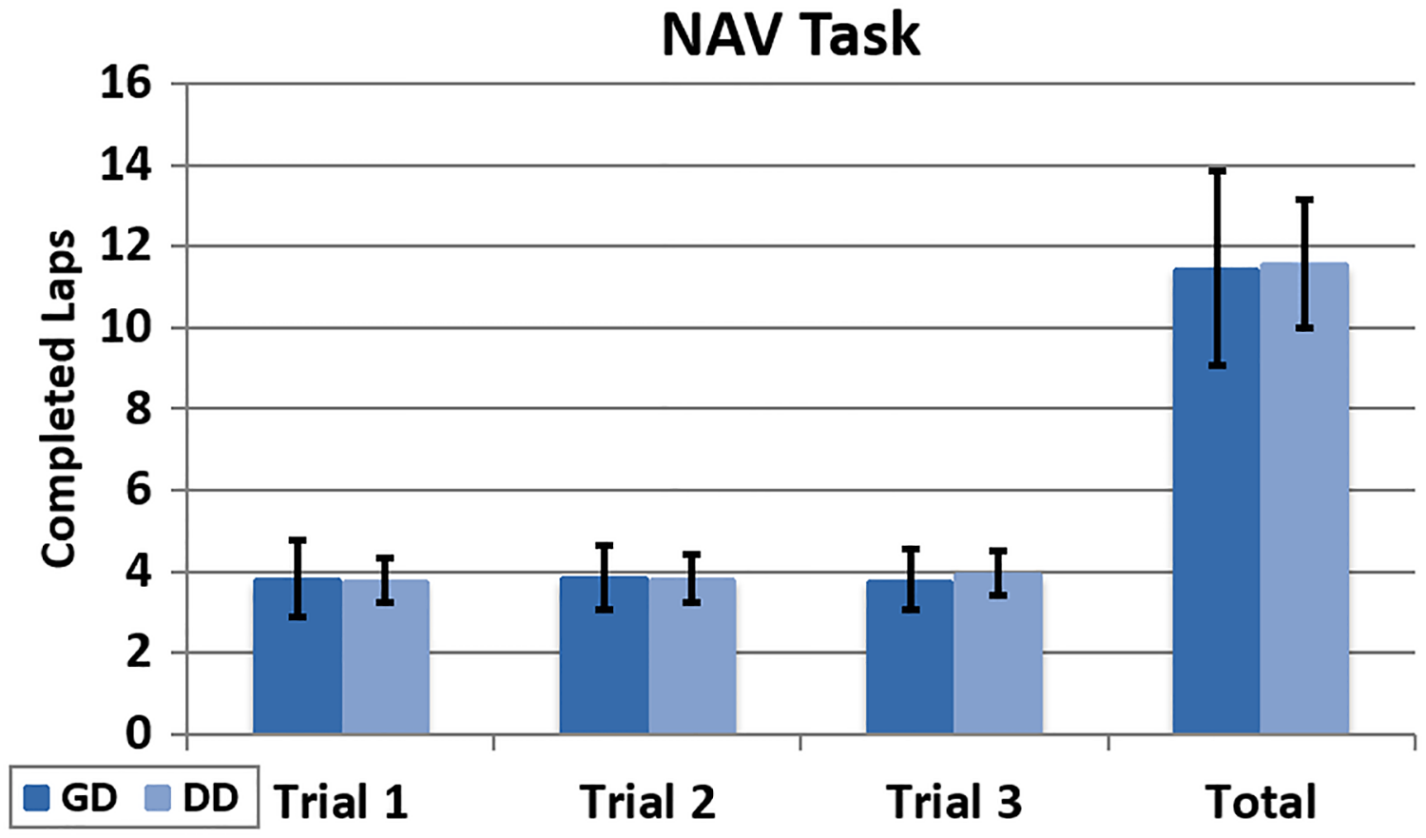
Completed laps (NAV). Completed laps per participant across trials during the NAV task.

The total number of completed laps per participant was very similar in the NAV task (Fig 15). The mean values were 11.5 and 11.6 for the GD and DD groups.

This result was expected and corresponds with the recorded deviations presented in Fig 10. The behaviour of the system is quite similar under the GD and DD conditions when the performed task involves navigation only.

**Fig 16:**
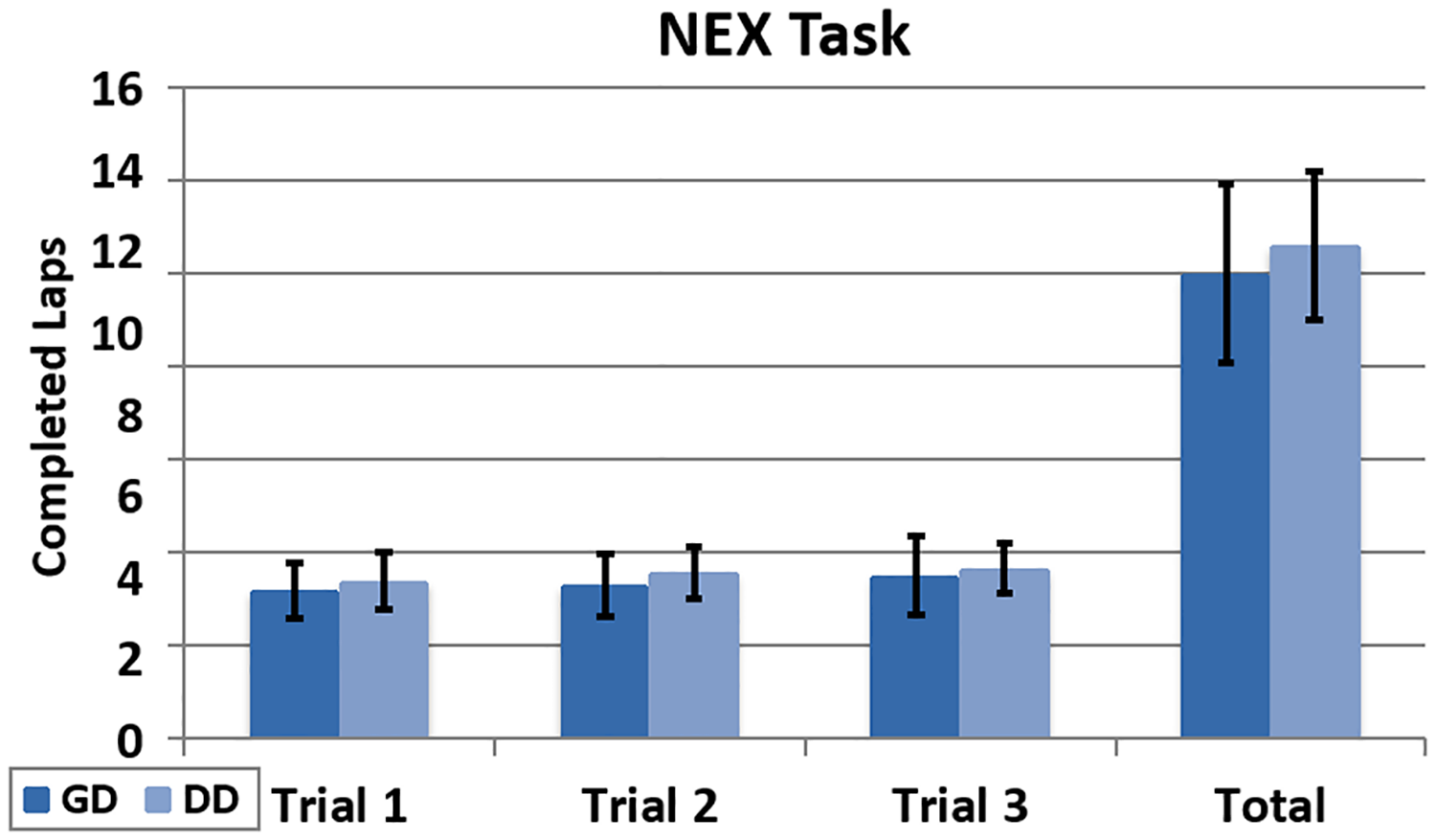
Completed laps (NEX). Completed laps per participant across trials during the NEX task.

During the NEX task, the total number of completed laps per participant was 10 in the GD group. This is 5.6% less than the 10.59 obtained in the DD group (Fig 16). However, this difference is not statistically significant (t(22) = 0.83, p = 0.41).

We did not expect this result. Because of the higher deviation from the ideal path that occurs during the NEX task under the GD condition, we expected that the participants would need to travel longer distances to complete a lap. Consequently, the number of laps under the GD condition would be lower in the NEX task. However, we observed that because of the deviation that causes the GD mode in the NEX task, some participants tended to go to the centre of the room. Therefore, they completed shorter laps and this increases the global average of completed laps per participant in the GD group. However, although statistically significant results are not obtained, it is worth mentioning that the number of completed laps is higher in all the trials under the DD condition.

**Fig 17:**
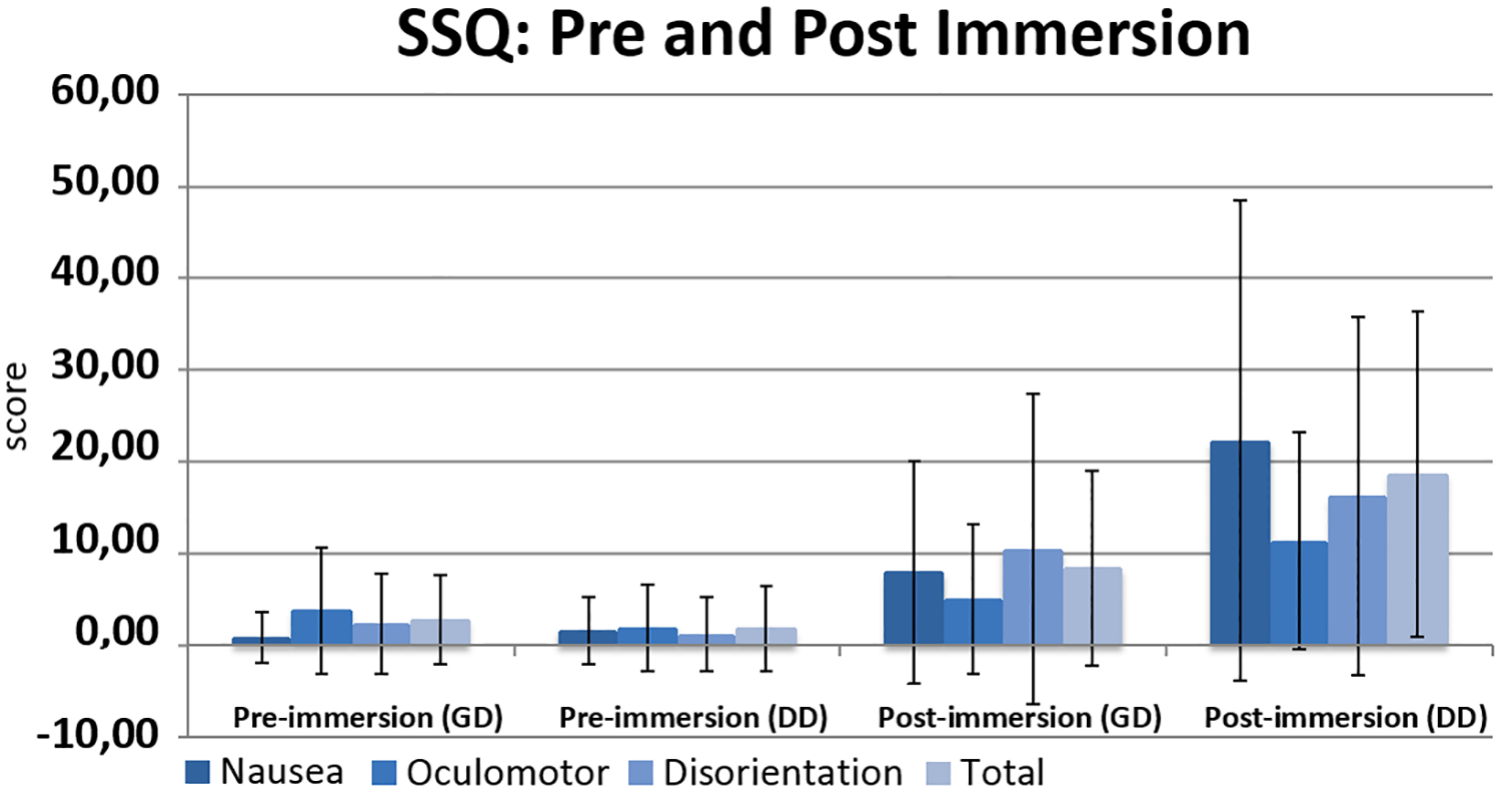
Results of SSQ (pre- and post-immersion).

We found that the registered score in the usability questionnaire was higher for the DD group (GD: M = 64.5, SD = 7.2; DD: M = 66.5, SD = 7.2). However, this difference is not statistically significant (t(22) = 0.671, p = 0.509). This is also the case for the presence questionnaire (GD: M = 45.3, SD = 6.9; DD: M = 48.1, SD = 6.05; t(22) = 0.907, p = 0.374) and the SSQ (Fig 17) in which all subscales obtained higher post-immersion values under the DD condition, although none of them is statistically significant (nausea: t(15,5) = 1.72, p = 0.11; oculomotor: t(22) = 1.52, p = 0.14; disorientation: t(22) = 0.78, p = 0.45; total: t(18.04) = 1.73, p = 0.1).

In the NAV task in which the participants were not affected by any distractions, the average number of completed laps is almost four. This gives a speed of 0.91 m/s, which still does not reach the normal walking speed but is closer to the normal speed value. The reason why the participants do not perform as they would in real life could be because the virtual reality in the experiments does not have all of the characteristics of a real experience. For example, the users cannot see their bodies and, as [2] points out, this fact can contribute to a decrease in the walking speed in the virtual environment.

Although this could strongly depend on the extent of technological development, real walking has been reported as a navigation technique that is prone to increasing cybersickness [52]. Thus, it is not clear to what extent each of the techniques, real walking and EHBD, contributes to increasing cybersickness.

We could observe during the experiment how wide the range of human tolerance to cybersickness is. While some people had to leave the study after a few steps, others started to jump and run on their own initiative during the adaptation phase. This is consistent with previous findings reported in [53].

Some participants from the GD group felt as if they were going to collide with the walls during the NEX task. Their reactions were identical to those expected in a real environment. Others completely forgot the real world during the study. These observations support the idea that natural locomotion contributes to an increase in the extent of involvement in the virtual experience.

Negative aspects collected in the general questionnaire were the latency of the system, resolution of the HMD and cybersickness caused by latency, searching for balloons and the limitation of the system while estimating the position of the head from sensors placed on the feet. Some of these comments were as follows: the slight display lag caused problems balancing sometimes; I felt tired of the image (visual fatigue); dizziness caused when moving the head too much; drift; sickness (at the beginning); gaps between pixels could be seen.

Some of the positive aspects highlighted for the behaviour of the system were how comfortable it is and high levels of immersion. Some of these comments were as follows: the system was very responsive to my movements; couldn’t really feel the equipment I was wearing; I found that the system is really impressive; it is a fantastic experience; I did feel as if I was there; I felt I was in another world.

Participants rated the system with an overall score of 7.3 out of 10 points. The GD condition scored slightly higher (7.7) than the DD condition (7.5) during the NAV task. This tendency was inverted during the NEX task, where GD achieved 6.9 points and DD 7.1.

The most frequent suggestion to improve the system was to include the avatar of the user in the experience. Several people tried to see their hands and feet during the study. Tracking of the hands and the addition of more interaction in the experience were also requested by some participants.

## Conclusions and future work

We have presented a wireless virtual reality system that provides a natural locomotion interface for navigation across virtual environments. The system is based on a reduced set of small inertial sensors. This approach contributes to reducing the cost of the system while making it comfortable and portable. We have addressed the difficulties derived from the decoupling of the body and head directions using inertial sensors indoors where magnetometers are not effective, leading to the appearance of drift errors in the yaw angle. To solve this problem, we have developed and presented the EHBD technique that was evaluated experimentally.

The learning effect is not observed across the tasks and the trials. It is found that the system is natural enough so that a quick adaptation phase is sufficient to make participants feel comfortable and obtain good results. According to the results, the EHBD technique allows effective decoupling in the directions of the body and the head while performing tasks that involve navigation only or navigation together with exploration. Most importantly, the results of the study are very promising: it fulfils the initial objectives and data traces and valuable feedback from the participants will contribute to improving the system. Furthermore, according to answers in the questionnaires, 100% of the participants who completed the experiment recommended the experience.

Regarding future work, the oscillation of the body around the vertical axis that can be observed while walking can be considered during the equalization process to improve the accuracy of the EHBD technique. For this purpose, the percentage of the gait cycle could be derived from the sensors on the feet [43]. As was suggested by several participants, including hand interaction and the use of inverse kinematics to display the avatar of the users will also improve the system.

## Supporting information

S1 File EHBD simulations. (XLSX)

S2 File Real walking experiment–results. (XLSX)
